# Globally Mobile Populations and the Spread of Emerging Pathogens

**DOI:** 10.3201/eid1511.091426

**Published:** 2009-11

**Authors:** Paul M. Arguin, Nina Marano, David O. Freedman

**Affiliations:** Centers for Disease and Prevention, Atlanta, Georgia, USA (P.M. Arguin, N. Marano); University of Alabama at Birmingham, Birmingham, Alabama, USA (D.O. Freedman)

**Keywords:** Emerging pathogens, globally mobile populations, bacteria, viruses, influenza, tuberculosis, chikungunya virus, severe acute respiratory syndrome, dengue, commentary

During the past decade, the global public health community has been challenged by the emergence and rapid worldwide spread of novel influenza strains, severe acute respiratory syndrome, chikungunya virus, drug-resistant tuberculosis, and other conditions and pathogens. Modern transportation and increased tourism, business travel, and immigration contributed to dissemination of these high-impact pathogens. The effectiveness of interventions such as airport screening, travel restrictions, and other community mitigation measures remains uncertain. However, human migration has occurred for centuries and will continue, despite the threats posed by microbes.

Medicine and public health traditionally have focused on the individual pathogens. Today, however, we should look more closely at globally mobile populations that move pathogens across international borders. In addition, we should consider what travelers’ behaviors, demographics, or geographic origins tell us about the microbial hitchhikers they might bring with them.

Travel and migration medicine are unique disciplines because of their dual focus on protecting the health of the individual and protecting the community in which that individual lives, works, or travels. Articles in this issue highlight globally mobile populations and stimulate thought about a recurring theme in travel and migration medicine: better identification and definition of at-risk travelers. We need to be able to identify these populations of travelers and characterize them appropriately so we can better identify modifiable risk factors and target interventions to keep travelers safe and healthy during and after their journeys.

Globally mobile population is a fairly broad, intentionally inclusive term. The fields of travel and tropical medicine usually are associated with preparing tourists for international journeys or evaluating such travelers when they return sick. Articles in this issue demonstrate a much broader concern because of the existence of many different types of globally mobile populations. This issue features articles on some of those populations: refugees, immigrants (legal and not), long-term travelers, pregnant travelers, guest workers, soldiers, cruise ship passengers, and imported animals (*1**–**6*). These extremely different populations share a characteristic: they travel from one part of the world to another, placing themselves or others at risk for exposure to novel conditions and pathogens that can adversely affect their health.

In addition to articles about host populations are articles about populations of microbes for which epidemiologic niches have been shifted by our globally mobile populations. For example, travel and migration affect the spread of antimicrobial drug resistance, vaccine-preventable diseases, multidrug-resistant tuberculosis, novel influenza viruses, and dengue virus serotypes (*7**–**9*). The risks of travel in developing countries are known; however, imported infection also can originate in wealthy countries and on luxury cruise ships (*5**,**10*). These observations, although perhaps intuitive, help establish the foundation of the evidence base for recommendations for travel and migration medicine.

Travel and migration medicine are still fairly young fields. Much of the medical literature, including the articles in this issue, still focus on defining populations and describing diseases and conditions associated with certain groups or activities. Relatively few of these articles recommend or evaluate new interventions to keep globally mobile populations safer and healthier. Investigators and public health authorities need to start making this shift towards scientific evaluation of interventions that can lead to using this evidence to begin shifting toward recommendations for efficient, cost-effective methods to prevent illness in refugees, immigrants, and travelers. At the same time, all disease- or pathogen-specific guidelines from national and supranational bodies should explicitly address globally mobile populations. Studies that measure the impact of pretravel guidance, vaccines, and prescription of prevention or self-treatment medications will then follow.

We have many lessons to learn from the increasing number of communicable diseases associated with transportation and travel. The traveling public is our teacher; let us take this opportunity to focus on the intersection between the travel and migration medicine and public health communities to improve the control and prevention of infectious diseases in globally mobile populations.

**Figure:**
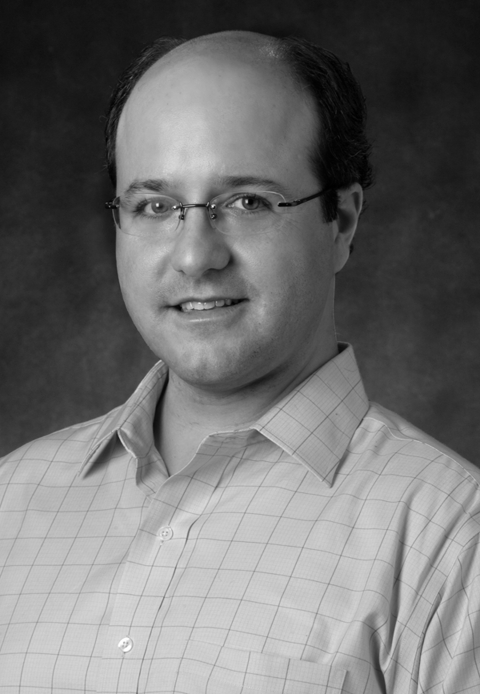
Paul M. Arguin

**Figure:**
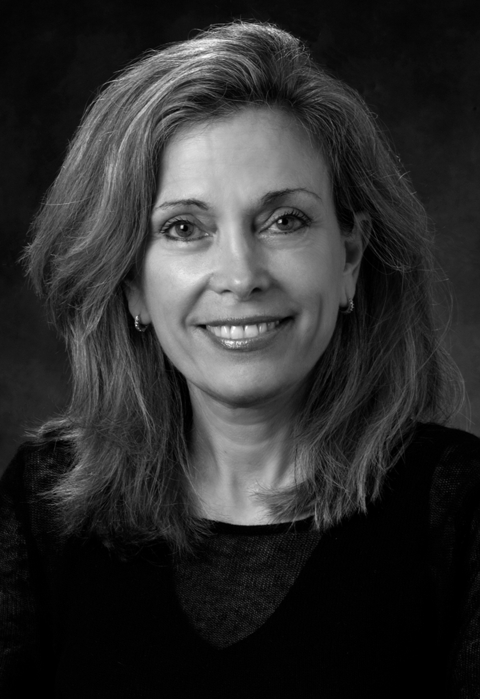
Nina Marano

**Figure:**
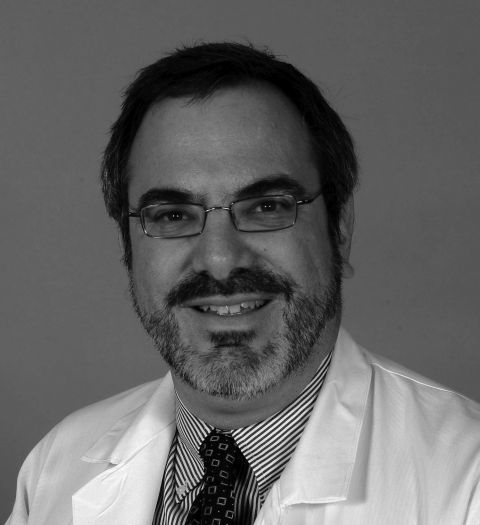
David O. Freedman
